# A machine learning-based predictive model of causality in orthopaedic medical malpractice cases in China

**DOI:** 10.1371/journal.pone.0300662

**Published:** 2024-04-17

**Authors:** Qingxin Yang, Li Luo, Zhangpeng Lin, Wei Wen, Wenbo Zeng, Hong Deng

**Affiliations:** 1 School of Forensic Medicine, Kunming Medical University, Kunming, China; 2 West China Hospital of Sichuan University, Chengdu, China; University of Macerata: Universita degli Studi di Macerata, ITALY

## Abstract

**Purpose:**

To explore the feasibility and validity of machine learning models in determining causality in medical malpractice cases and to try to increase the scientificity and reliability of identification opinions.

**Methods:**

We collected 13,245 written judgments from PKULAW.COM, a public database. 963 cases were included after the initial screening. 21 medical and ten patient factors were selected as characteristic variables by summarising previous literature and cases. Random Forest, eXtreme Gradient Boosting (XGBoost) and Light Gradient Boosting Machine (LightGBM) were used to establish prediction models of causality for the two data sets, respectively. Finally, the optimal model is obtained by hyperparameter tuning of the six models.

**Results:**

We built three real data set models and three virtual data set models by three algorithms, and their confusion matrices differed. XGBoost performed best in the real data set, with a model accuracy of 66%. In the virtual data set, the performance of XGBoost and LightGBM was basically the same, and the model accuracy rate was 80%. The overall accuracy of external verification was 72.7%.

**Conclusions:**

The optimal model of this study is expected to predict the causality accurately.

## 1. Introduction

Machine learning (ML) is subfield of artificial intelligence (AI) that focuses on teaching computers to identify and interpret patterns within data through training [1]. ML have demonstrated potential across various domains within the biomedical sciences, such as genomics [2, 3], clinical medicine [4, 5] and forensic medicine [6, 7]. Models that have been published in clinical medicine can enhance the alertness of clinicians, carry out diagnostic procedures, predict events pertinent to clinical practice, and steer the process of making clinical decisions [8, 9]. However, few models have been used in clinical practice, which may be due to the challenges of machine learning models in feature selection, model complexity and generalization ability, the quantity and quality of training data, model interpretability, and other ethical and legal factors [10]. Within the realm of forensic medicine, the implementation of AI has the potential to augment the capabilities of human experts, effectively mitigating the inherent subjectivity and bias associated with conventional forensic methodologies. Forensic anthropology primarily involves the reconstruction of biological profiles for deceased individuals to ascertain their identity, including attributes such as sex, age at the time of death, and ancestry [11, 12]. In forensic odontology, AI has been utilized to forecast age and gender from dental characteristics, facilitating both human identification processes and the analysis of bite marks [13]. In disability assessment, researchers have tried to combine ML with the International Classification of Functioning, Disability, and Health (ICF) to assess the degree of disability more accurately and conveniently [14, 15]. In addition, ML also has some applications in forensic pathology, forensic genetics and other forensic branches. But it is scarcely any used in medical malpractice.

In recent years, the number of medical malpractice tort liability cases in China has increased, and orthopaedics is one of the departments with the most cases [16, 17]. Due to medical disciplines’ high professionalism and complexity, judges need to rely on professional technical assistance to adjudicate such cases. In order to ensure fairness and justice of verdicts, China has established a "dual-mode" structure of two third-party authentication organisations, the medical association identification and the judicial appraisal institution, which is similar to the system of single joint expert (SJE) [18]. The medical association identification and the judicial appraisal institution do not represent any of the parties and have a neutral status. They accept the commission of the court by experts to conduct a retrospective analysis of the cases and then issue a written appraisal opinion which will become scientific evidence [19].

The identification of medical malpractice cases in China needs to consider four issues: (1) Whether there is any fault in the medical treatment process; (2) Whether the doctor has caused substantial harm to the patient; (3) Whether there is a factual causality between the physician’s fault and the patient’s damage; (4) The degree of the factual causality. Experts can make scientific identifications based on textbooks, clinical guidelines and etc., to determine whether there is any fault in the medical treatment process. However, as for the degree of factual causality, how to judge it scientifically is still the slip of everyone’s debate. Scholars have put forward many different theories, such as "Bolam standard", "Forcier-Lacerte medicolegal causal analysis model" [20], "Integration of Forensic Epidemiology and the Rigorous Evaluation of Causation Elements (INFERENCE)" [21]. Many causal analysis methods are being applied in various countries, and experts inevitably mix subjective factors in practical application. Especially in the face of complex causal issues, the opinions of different experts in the same case may be different [22]. This makes the objectivity and reliability of the identification opinions questionable [23]. Therefore, improving the reliability of identification opinions and reducing subjective factors has become an urgent issue that needs to be solved.

**Fig 1:**
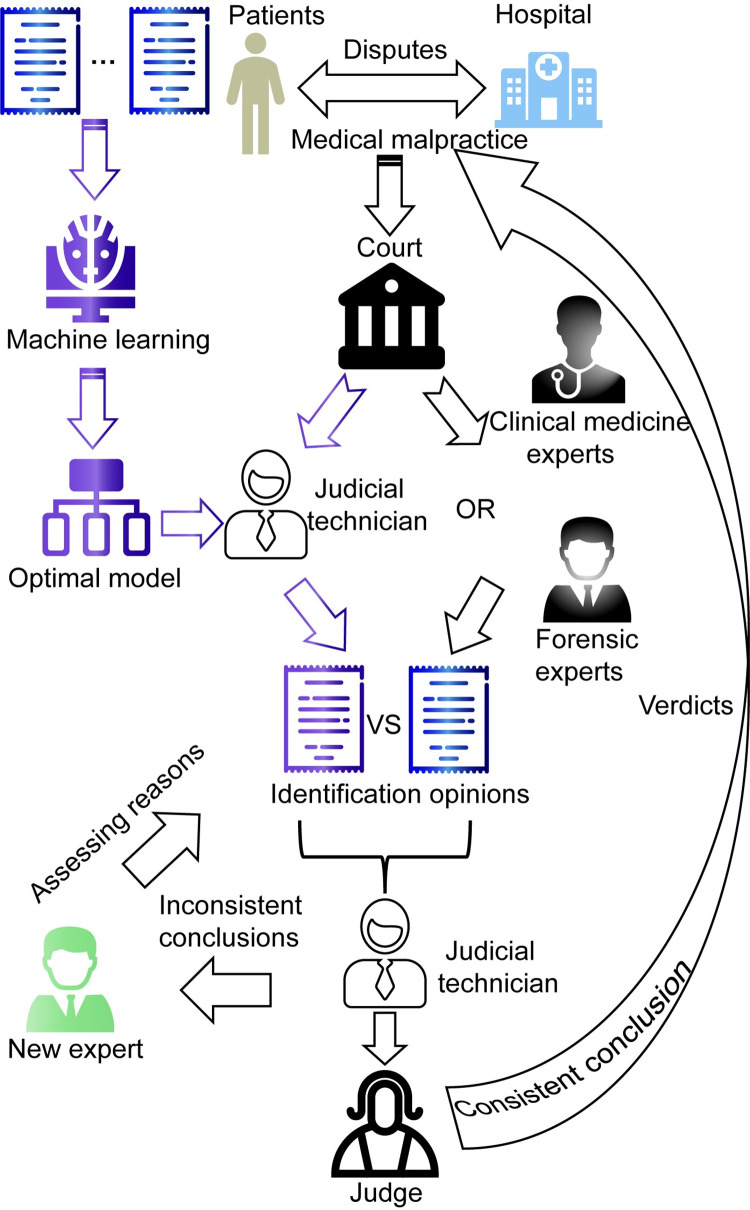
The new decision-making process of medical malpractice.

In China, the conventional procedure for medical malpractice tort liability cases typically unfolds in a structured sequence: Initially, a dispute arises between the patient and the hospital, prompting both parties to jointly initiate legal proceedings in court. Subsequently, the court entrusts the case to a medical association, composed of clinical medicine experts, or a judicial appraisal institution staffed with forensic experts for an impartial evaluation. These experts meticulously examine the case and formulate their identification opinions, which are then submitted to the judicial technician for thorough review. The technician’s assessment is ultimately conveyed back to the judge, who renders a verdict based on the expert findings and the merits of the case. Even though the forensic expert is highly trained and uses specific guidelines, subjectivity can lead to inaccuracies in the evaluations, for example, errors due to incorrect analysis of the data available or the methodology followed in complex cases. Therefore, we present a new process that shows how machine learning can be integrated into decision-making process to reduce the margin of error in evaluation (Fig 1). We commence by leveraging a dataset comprising medical malpractice cases within the field of orthopedics in China as our foundational training set. We meticulously curate medical and patient factors as key features and harness the power of machine learning to construct an optimal causality prediction model. Building upon this traditional methodology, we introduce an innovative process: upon the court’s delegation of a case to expert evaluators, the case details are meticulously processed through feature extraction and fed into our premier machine learning model. Subsequently, the judicial technician meticulously compares the model’s classification outcomes with the expert’s assessments. Should the findings align, the expert’s opinion is deemed highly reliable, thereby informing the judge’s decision-making process. Conversely, in the event of discordant conclusions, the court must engage a new expert to scrutinize the inconsistencies and provide a cogent explanation, ensuring the integrity of the judicial process.

**Fig 2:**
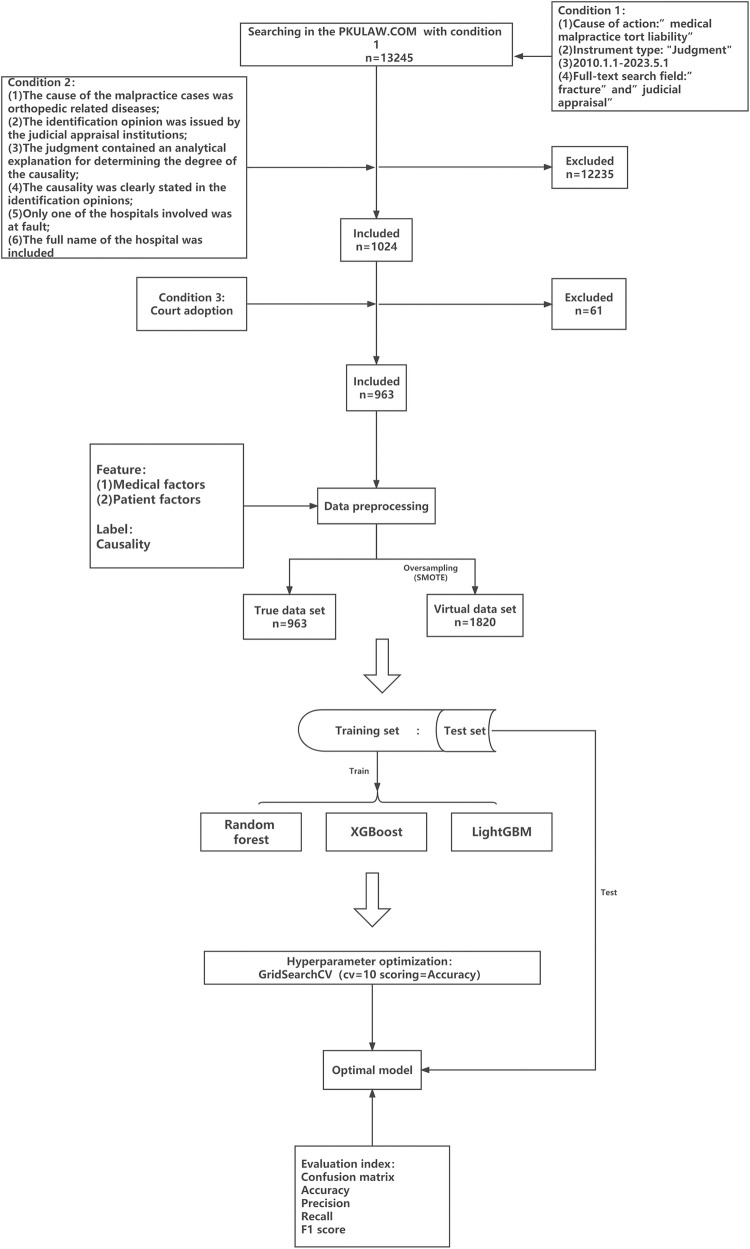
The complete flow chart of this study.

In this way, our purpose is to propose a ML supplementary means based on real case data, aiming to explore the feasibility and validity of machine learning models in determining causality in medical malpractice cases and to try to increase the scientificity and reliability of identification opinions. as well as ultimately improve the fairness of court verdicts. The complete flow chart of this study is shown in Fig 2.

## 2. Material and methods

### 2.1 Source of data

**Firstly**, it was searched in the PKULAW.COM (a public database) under the following conditions: (1) Cause of action: "medical malpractice tort liability"; (2) Instrument type: "Judgment"; (3) 2010.1.1–2023.5.1; (4) Full-text search field: "fracture" and "judicial appraisal". A total of 13,245 cases were retrieved. **Secondly**, the following additional criteria were included: (1) The cause of the malpractice cases was orthopaedic-related diseases; (2) The judicial appraisal institutions issued the identification opinion; (3) The judgment contained an analytical explanation for determining the degree of the causality; (4) The causality was clearly stated in the identification opinions; (5) Only one of the hospitals involved was at fault; (6) The full name of the hospital was included. We filtered the case contents one by one according to condition. 1024 cases were included, while 12235 cases were excluded. **Finally**, based on whether the identification opinions were admissible in the final judgment, 963 cases were included, while 61 cases were excluded from the total of 1024 cases. All the data in this study are from a public database, which does not contain any privacy-related information after processing, so there is no need for an ethical review.

### 2.2 Feature selection and data preprocessing

The label was "Causality". Guidance for judicial expertise of medical malpractice (China SF/T 0097–2021) [24] classified causality into six degrees: (1) No causality: The consequences of damage were almost entirely due to patient factors, and there is no essential correlation with medical behaviour. (2) Minor causality: most of the damage consequences were due to patient factors, and the medical factors induced or slightly promoted and aggravated the effects. (3) Secondary causality: The damage consequences were primarily due to patient factors, and the medical factors played a role in promoting and aggravating. (4) Equal causality: Medical factors and patient factors played similar roles in forming damage consequences, and it was difficult to distinguish the primary and secondary. By parity of reasoning, there was the main and whole causality.

Thabet et al. ’s [25] meta-analysis of orthopaedic litigation divided the factors causing litigation into medical factors (diagnostic faults and procedural faults) and patient factors (nature and location of injury). Dong et al. ’s [26] graph theory analysis study established a complex network of medical malpractice in China, in which factors such as the technical and non-technical faults of the medical provider, the type of disease of the patient, and the degree of damage caused by the medical provider to the patient have their respective proportions.

We summarized the previous literature reports [14, 25–31], after fully understanding those common factors and considering the reality of medical malpractice cases in China, and divided the influencing factors on the degree of causality into medical factors (91 technical faults and 29 non-technical faults) and 10 patient factors (S1 Table). Regarding the attribution of medical malpractice, it is generally observed that an increased number of affirmative responses in medical factors correlates with a higher degree of hospital liability in the case. Conversely, a greater number of affirmative responses in patient factors typically diminish the proportion of medical responsibility. However, exceptions to this general rule may arise when the hospital’s negligence results in exceptionally severe consequences, or when the physician’s error, though entirely preventable, was inescapable. To navigate these complexities, we employed a hybrid approach, integrating data-driven insights with domain expertise. This methodology involved extensive consultation with five forensic and five clinical experts, culminating in the identification of a refined set of 31 characteristics for further analysis. **Medical factors**: (1) Hospital level; (2) Missed diagnosis and delayed treatment; (3) Inadequate preoperative preparation; (4) Insufficiency of operative pointer; (5) Inadequate therapeutic schedule; (6) Inadequate alternative treatment; (7) Inadequate operation technique; (8) Inadequate manual reduction; (9) Inadequate external fixation; (10) Inadequate internal fixation; (11) Anesthesia problem; (12) Inadequate nursing and observation; (13) Inadequate postoperative examination; (14) Inadequate medication use; (15) Insufficient recognition; (16) Inadequate hospital management; (17) Inadequate discharge instructions; (18) Inadequate contingency handling; (19) Inadequate consultation and referral; (20) Inadequate informed consent; (21) Medical record problem. **Patient factors**: (1) Over 60 years old; (2) Traumatic or not; (3) Number of other diseases (Such as diabetes, osteoporosis, nutritional status, etc.); (4) Comminuted fracture or not; (5) Number of hospitalisations; (6) Damage consequence (Divided into: no, prolonged course of disease, aggravated disease, disability, death); (7) Lack of compliance; (8) Severe illness or progress rapidly; (9) Uncommon disease; (10) Prognosis of disease. Crafting an effective dataset necessitates the meticulous identification of a cadre of salient, quantifiable factors and the development of unbiased scales that enable each unique case to be distilled into a standardized array of descriptors. Given the intricate interplay of medical and legal expertise inherent in Chinese medical malpractice adjudications, conventional natural language processing (NLP) techniques fall short in capturing the pertinent details. Consequently, we have adopted a meticulous manual reading approach to feature extraction, ensuring the fidelity and nuance of the data are preserved. Some features were grade variables, while the rest were binary variables. The above features and label assignments are shown in Table 1. By converting the information in the judgment into characters, and then calculating the common features by the ML algorithm, it is presented as a prediction model for the causality.

**Table 1:** Features and label assignment.

Name	Assignments
Causality	No = 0; Minor = 1; Secondary = 2; Equal = 3; Main = 4; Whole = 5;
Damage consequence	No = 0; Prolonged Course of disease = 1; Aggravated disease = 2; Disability = 3; Death = 4;
Hospital level	No = 0; First = 1; Second = 2; Third = 3;
Number of other diseases	No = 0; One = 1; Two = 2; Three or more = 3;
Number of hospitalisations (median = 2)	Less than or equal to 2 = 0; Greater than 2 = 1;
Medical factors Patient factors	No = 0; Yes = 1; No = 0; Yes = 1;

### 2.3 Model selection and establishment

**Fig 3:**
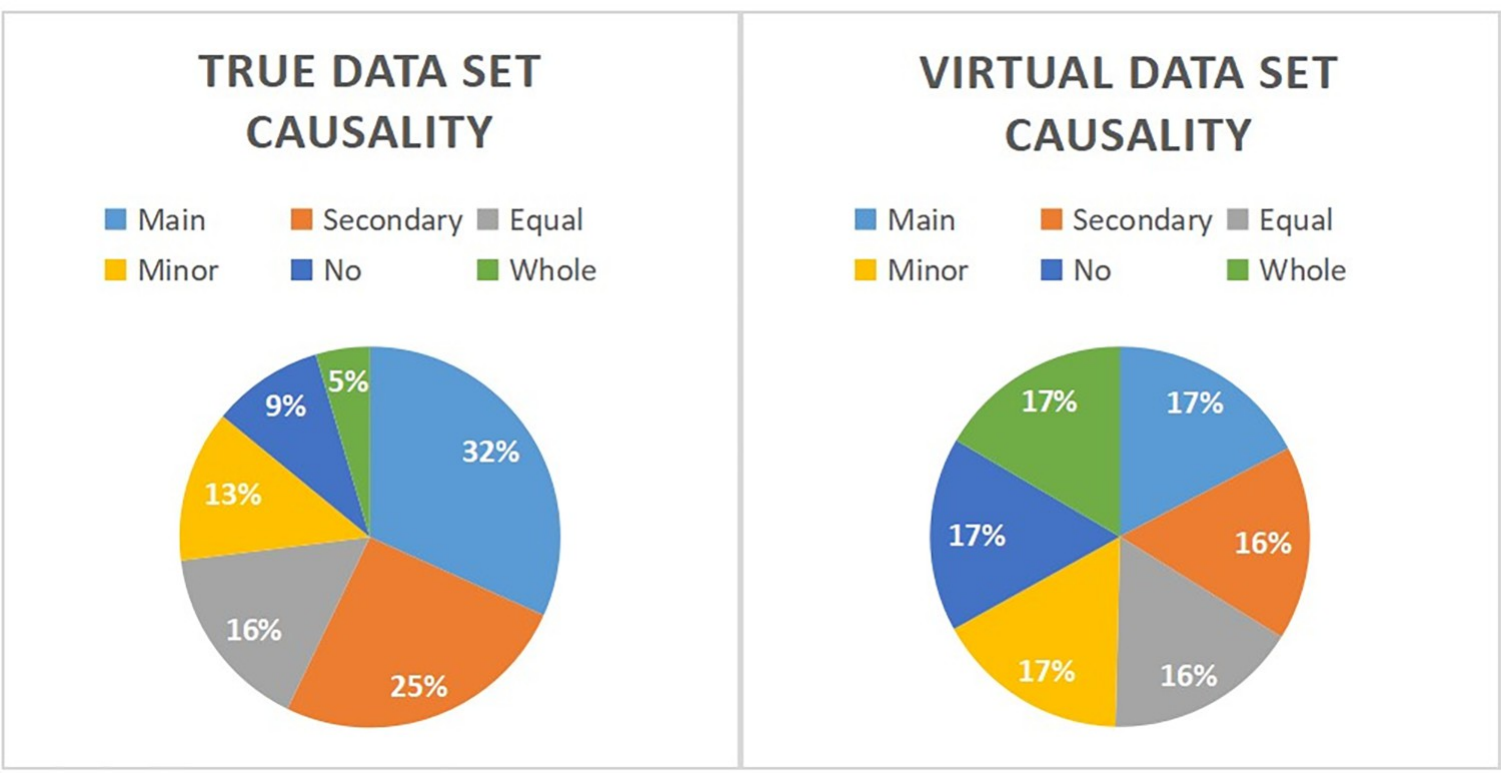
Label "Causality" proportion of each category.

The 31 feature variables and one label selected in the true data set of this study were all classified variables after preprocessing, and the categories of labels, as shown in Fig 3, belong to the labelled imbalanced data set. Our goal was to train the model based on the training set and then accurately classify and predict the test set based on the model. Therefore, this paper chose three Ensemble Learning models based on the Decision tree model in machine learning classification algorithm: Random Forest, XGBoost, and LightGBM [32]. Decision Tree was a flowchart-like tree structure where each internal node denotes a test on an attribute, each branch represents an outcome of the test, and each leaf node (terminal node) holds a class label. These three models can sample according to weights to balance the test set data, perform well for imbalanced multi-classification problems, and output the importance of features, which has guiding significance for the subsequent feature selection. In addition, machine learning algorithms based on decision tree-based models also have the advantages of less data preparation, no data normalization, no data scaling, and missing values do not affect the modeling process. Last but not least, the tree model is highly interpretable, and the black-box problem was solved by output tree structure.

Python 3.10.7; Compiler: Jupyter Notebook were used as the language environment of this study. First, we imported the python base function library including "numpy", "scipy", "pandas"and the drawing function library containing "matplotlib.pyplot", "seaborn". Then, we divided the real data set into the training and test sets with a weight ratio of 7:3, "random_state" = 6, using train_test_split from sklearn.model_selection. We calculated Random Forest using RandomForestClassifier from sklearn.ensemble, XGBoost using XGBClassifier from xgboost.sklearn and LightGBM using LGBMClassifier from lightgbm.sklearn. After training the three models with the training set, the Grid Search Cross Validation (GridSearchCV, cv = 10, scoring = accuracy) was used to optimise the model hyperparameters. As for Random Forest, its hyperparameters had "n_estimators: range (10, 300, 10)", "min_samples_split: range (5, 50, 5)", "min_samples_leaf: range (2, 40, 2)", "max_depth: range (1, 30, 2)", "criterion: ’gini’, ’entropy’ ", "class_weight: None, ’balanced’ ". As for XGBoost, "max_depth: [3, 5, 7] ", "learning_rate: [0.1, 0.01, 0.001] ", "subsample: [0.1, 0.01, 0.001] ", "colsample_bytree: [0.5, 0.7, 1] ", "gamma: [0, 0.1, 0.2, 0.3, 0.4] ", "reg_alpha: [0, 0.001, 0.005, 0.01, 0.05] ", "reg_lambda: [0, 0.001, 0.005, 0.01, 0.05] ". The hyperparameters of LightGBM was same as XGBoost except "gamma". GridSearchCV exhaustively searches for all possible combinations in a given parameter space, then evaluates the performance of each combination, and finally selects the parameter combination with the best performance. Finally, the real optimal model was obtained. At the same time, to form a virtual data set with a labelled ratio of 1:1:1:1:1:1 (Fig 3), the Synthetic Minority Oversampling Technique (SMOTE), an oversampling method from imblearn.over_sampling, was used to expand the true data set. The virtual data set was divided into the training set, and the test set with 9:1 [32], "random_state" = 6, and then the virtual optimal model was established according to the above method.

### 2.4 Model performance evaluation

For multiple classification problems, the confusion matrix and the following four indicators were adopted as the criteria to measure the overall performance of the model:(1) Accuracy; (2) Precision; (3) Recall; (4) F1 score. True Positive (TP): A positive example of being correctly predicted. That is, the true value of the data was a positive example, and the predicted value was also a positive example. True Negative (TN): Counter-examples that their true data value was a counter-example, and the predicted value was also a counter-example. False Positive (FP): Positive example of misprediction. That is, the true value of the data was a negative example, but it was incorrectly predicted to be a positive example. False Negative (FN): A counter-example of being incorrectly predicted, in which the true value of the data was a positive example but incorrectly predicted to be a negative example [33].


$$Accuracy=\frac{TP+TN}{TP+FP+TN+FN}$$

$$Precision=\frac{TP}{TP+FP}$$

$$Recall=\frac{TP}{TP+FN}$$

### 2.5 Feature importance ranking

We used the attribute "feature_importances_" to see the importance of the feature.

### 2.6 External data validation

We collected 11 orthopaedic medical malpractice cases from a judicial expertise centre in Yunnan Province in 2021–2022 as an external data set to verify the performance of the best model.

## 3. Results

The 963 cases included in this study involved several hospitals and judicial appraisal institutions in 29 provinces all over China. There are 21 factors of doctors and ten factors of patients.

### 3.1 True data set

**Fig 4:**
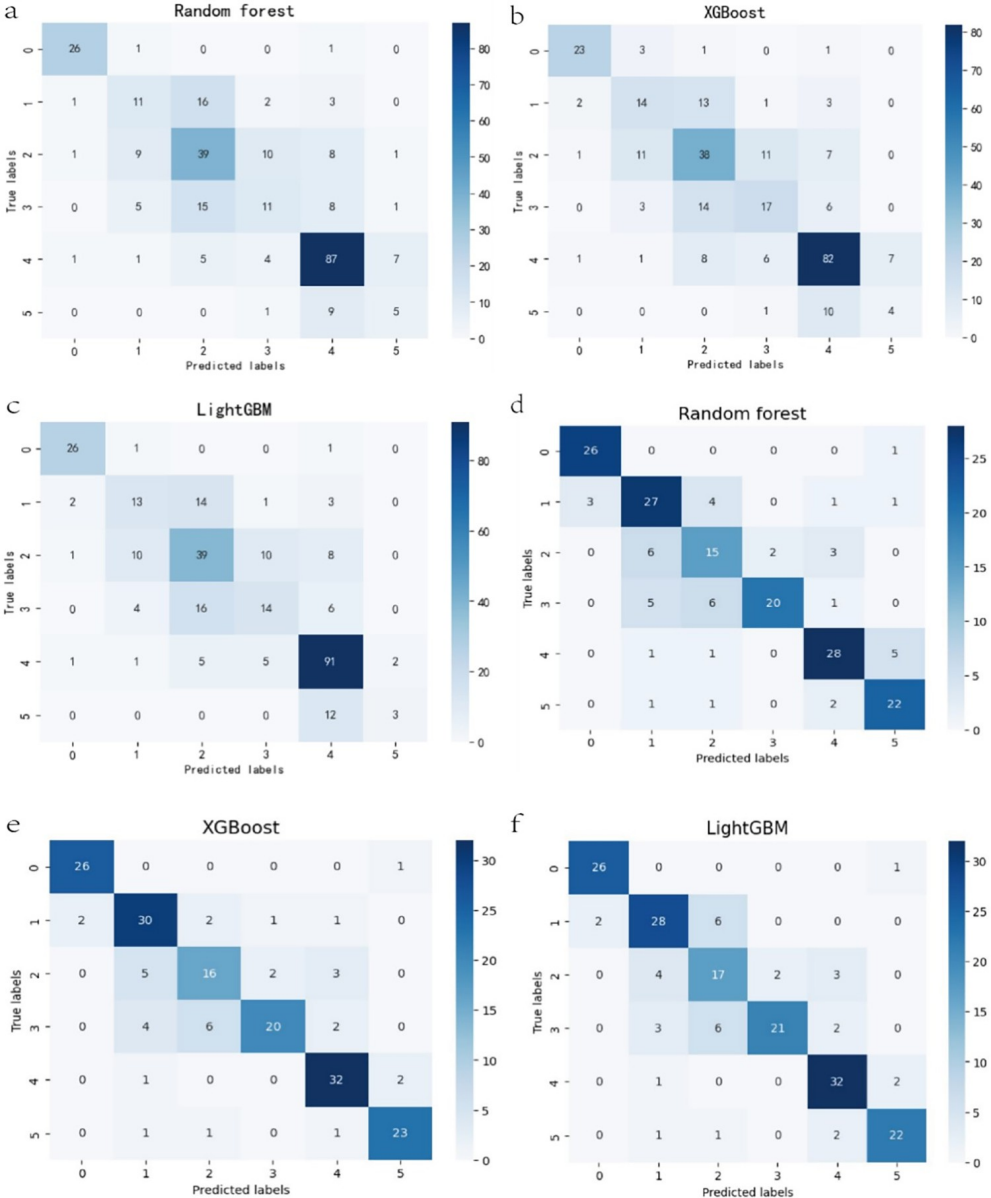
(a, b, c) The test set confusion matrix of the true data set. (d, e, f) The test set confusion matrix of the virtual data set. (“Predicted Label”: No = 0; Minor = 1; Secondary = 2; Equal = 3; Main = 4; Whole = 5).

The true data set was divided into the training set and the test set according to 7:3. That is, 289 cases were taken as the test set and put into the optimal prediction model of the three algorithms, respectively. The confusion matrix is shown in Fig 4. Among the three models, the precision of label classification as "No" is the highest, reaching 90%; The precision of "Main" is higher, reaching 70%-75%; The precision of "Minor", "Secondary", and "Equal" is slightly worse, at 40–55%; The precision of "Whole" is only about 40%, which is related to the small number of "Whole" in the true data set.

### 3.2 Virtual data set

The virtual data set of n = 1280 was formed by oversampling with SMOTE and divided into training sets and test sets according to 9:1. That is, 128 cases were put into the optimal prediction model of the three algorithms, respectively. The confusion matrix is shown in Fig 4. Among the three models, the precision of "No" and "Main" was the highest, which can reach more than 90%. The precision of "Minor", "Equal", and "Whole" was higher, reaching 70%-85%. "Secondary" precision was the worst, at about 55%.

### 3.3 Model overall performance comparison

Accuracy, Precision, Recall and F1 score were used to evaluate the model’s overall performance. The higher the value, the better the model performance. The comparison shows that the XGBoost model performed best in the real data set (Table 2). The performance of XGBoost and LightGBM in virtual data sets was better than that of Random Forest. In addition, the same algorithm performs better in virtual data set than in true data set. However, because the virtual data set was formed by oversampling, there may be problems with overfitting and data leakage.

**Table 2:** The performance of three models in true and virtual data sets. (RF: Random Forest; XGB: eXtreme Gradient Boosting; LGBM: Light Gradient Boosting Machine.).

	The true data set			The virtual data set		
	RF	XGB	LGBM	RF	XGB	LGBM
Accuracy	0.62	0.66	0.64	0.76	0.80	0.81
Precision	0.60	0.64	0.63	0.77	0.80	0.80
Recall	0.62	0.66	0.64	0.76	0.80	0.80
F1 score	0.61	0.63	0.63	0.76	0.80	0.80

### 3.4 External data validation

Eleven orthopaedic medical malpractice cases from a judicial expertise centre in Yunnan, China, were preprocessed and imported into an optimal model trained from a virtual dataset using XGBoost. The overall accuracy of the model was 72.7%. The results are shown in Table 3.

**Table 3:** Comparison of external validation results.

True value	0	3	1	4	4	3	3	1	4	2	5
Predicted value	0	3	2	4	4	2	3	1	4	2	4

### 3.5 Model interpretability and feature importance

ML algorithms contend with the issue of opacity, where the system fails to offer any coherent rationale or satisfactory elucidation for its decisions, a conundrum often referred to as "the black-box dilemma." The enigmatic character of ML algorithms poses a significant comprehension challenge for human understanding [34, 35]. Entrusting crucial decisions to a black-box model created a necessary need for ML algorithms to be explainable for their decision-making process [36]. Therefore, we chose the ML based decision trees which solved the "black box" problem by integrating the prediction results of multiple decision trees. Each decision tree was a "white box" that could be understood and interpreted. When these decision trees were integrated together, random forests could provide more stable and reliable predictions while maintaining the explanatory properties of individual decision trees. The model is capable of assigning an importance score to each feature, which serves as a valuable tool for elucidating the intricacies of the model’s predictive process. Furthermore, random forests are adept at uncovering interactions and nonlinear dynamics between features, thereby offering a more holistic and nuanced interpretation of the underlying data relationships. But the ML based decision trees construction process was done automatically, and the model learns and generates decision trees on its own based on the provided data, so there was usually no need for additional human opinion or intervention. The quality of the model depended on feature selection and hyperparameter optimization which can be manually intervened.

**Fig 5:**
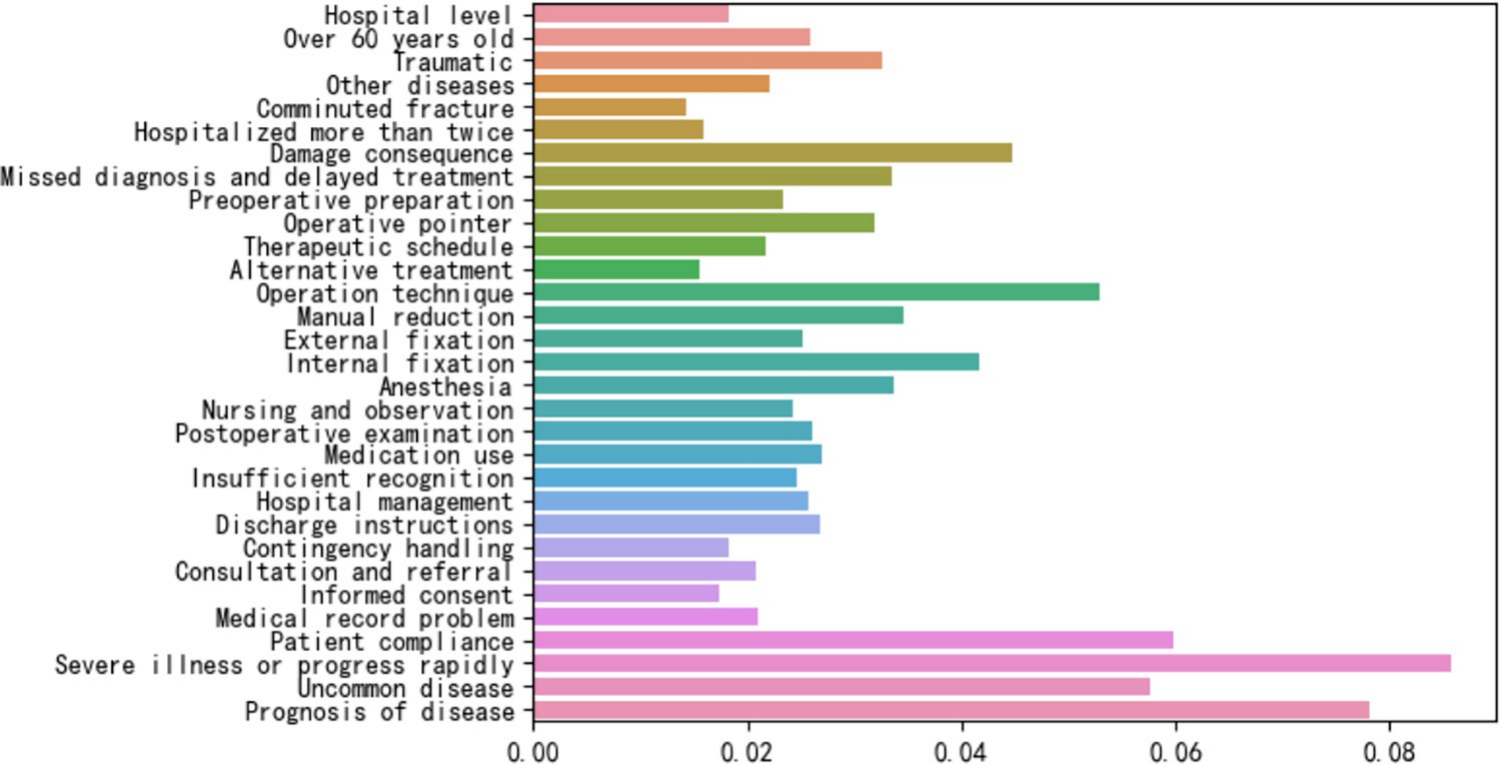
The feature importance of XGBoost, the optimal model for true data set.

Knowing the feature importance to the prediction model helped us better understand the decisions and actions of the model [37]. The feature importance of XGBoost in the optimal model of the true data set is shown in Fig 5. The top 10 features are: (1) Severe illness or progress rapidly; (2) Prognosis of disease; (3) Lack of compliance; (4) Uncommon disease; (5) Inadequate operation technique; (6) Damage consequence; (7) Inadequate internal fixation; (8) Inadequate manual reduction;(9) Anesthesia problem; (10) Missed diagnosis and delayed treatment. Among the three models, five items were ranked in the top ten: (1) Severe illness or progress rapidly; (2) Prognosis of disease; (3) Inadequate operation technique; (4) Damage consequence; (5) Inadequate internal fixation.

## 4. Discussion

In the true data set of this study, the distribution of labelled "Causality" was similar to the normal distribution, and relatively few cases were classified as "No" and "Whole". There were a few cases in which the court ruled that there was no causality or whole causality between medical factors and patient damage consequences. The data set’s imbalance nature led to a decline in the accuracy of machine learning models, with the optimal model only reaching 66%. However, when we used the oversampling method to form a virtual balanced data set, the model’s accuracy increased significantly to 80%. To verify the real performance of the model, we used 11 cases as an external dataset for external validation, and its overall accuracy was 72.7%, close to the ideal 80%. This data set only collected cases of orthopaedic-related medical malpractice, and the model can only be used in orthopaedic departments. However, the accuracy of 72.7% indicates that the machine learning algorithm can accurately predict the degree of causality in complex causation cases when the sample size of the dataset is large enough. We can predict the degree of causality of medical malpractice associated with other departments by re-selecting features and datasets.

Feature selection plays an essential role in model accuracy [38]. Regarding feature selection, we believe there are essential differences between different clinical departments in terms of medical factors, especially in technical issues [39]. At the same time, the different patient has different patient factors. Therefore, this study only included the data of orthopaedic-related medical malpractice cases and specialised and detailed the factors of doctors and patients as much as possible [25]: (1) Severe illness or progress rapidly; (2) Prognosis of disease; (3) Inadequate operation technique; (4) Damage consequence; (5) Inadequate internal fixation. The above five features are ranked in the top ten of the three models, which can inspire us. Orthopaedic surgeons need to pay more attention to technical issues, similar to the study by Liu et al. [40]. Of course, the patient’s disease and development are also essential factors affecting responsibility identification.

At present, the determination of liability for medical malpractice relies on identification opinions, and the adoption rate of identification opinions by courts is very high [41], which is as high as 93.7%, according to the data in this paper. When issuing identification opinions, the experts’ cognition of causality in the same case is biased due to their own background or experience [42, 43]. Ultimately, the identification opinions obtained by different experts in the same case may differ greatly, but it does not mean the different identification opinions are wrong. In order to standardise the identification opinions of medical malpractice evaluation, countries have drawn up a series of guidelines, such as "Guidance for judicial expertise of medical malpractice" (China SF/T 0097–2021) [24]; "The European Guidelines on medicolegal Methods of Ascertainment and Criteria of Evaluation" [44]. However, these guidelines do little to bridge the difference between identification opinions on the same case. Advanced computer technology has been widely used in forensic anthropology, dentistry and other disciplines [7, 45]. We expect to reduce cognitive bias through machine learning algorithms and models based on many Chinese case data. For example, in an orthopaedic medical malpractice case, after the expert has reached an identification opinion (causality), the case-related information (feature) is imported into the model, and the model predicts the most likely degree of causality (label) through a series of calculations. If the two are consistent, the expert identification opinion is more reliable.

The lack of medical and forensic knowledge puts judges in a difficult position when reviewing identification opinions [46]. The current solution is for experts to write their reports in plain language [47], or have the experts appear in court to explain their reports [48]. This study provides a new way to review identification opinions based on case data and computer algorithms. Machine learning can provide prediction and assessment of case outcomes, assist judges and lawyers to make more accurate decisions and reduce the influence of subjective factors. By reducing the interference of human factors, machine learning can improve the fairness and objectivity of court decisions and ensure the impartiality of justice. In particular, the data in this study comes from written judgement in a publicly available database. However, due to privacy issues and the fact that the court does not require the extraction of expert identification opinions in the judgment, and the effective information in the public database is limited, only 963 qualified cases are screened out of 13,245 judgments. More data for the courts, which have complete data on expert opinions, means that more accurate models can be built. Before this, judges could not judge whether the expert opinions made by the commissioned experts were reasonable. Now, the machine learning prediction model formed by the modeling of a large amount of data can represent the mainstream opinion of most experts to a certain extent, which provides a basis for judges to compare. In this way, judges are no longer bogged down with a lot of medical knowledge and only need to compare the results of models with those of experts. If they are different, more scrutiny is needed.

The use of machine-learning algorithms in the justice system involves multiple ethical issues that require careful consideration in the deployment and use of these technologies [49, 50]. ML algorithms may inadvertently learn and amplify existing social biases, leading to unfair treatment of certain groups of people, such as those of a particular race, gender, or socioeconomic status [51]. So we did not include sex, race, and ethnicity in the characteristics, even though they might be important to the model. In addition, there is a growing demand to be able to "explain" ML systems’ decisions and actions to human users, particularly when used in contexts where decisions have substantial implications for those affected and where there is a requirement for political accountability or legal compliance [52]. As mentioned earlier, we have adopted methods with high explanatory power for modeling. What’s more, ML in the justice system need to process large amounts of sensitive data, including personally identifiable information, criminal records, and more. How to ensure the security and privacy of these data and prevent data leakage and misuse is an important ethical challenge. Besides, when machine-learning algorithms play a role in judicial decision making, how can responsibility be assigned if errors occur? Clear regulatory and legal frameworks for the use of machine learning in the justice system are lacking. This can lead to a lack of appropriate oversight and accountability mechanisms in practice. These issues remain to be resolved.

ML methodologies can be harnessed to categorize disability levels, employing a comparable approach to amass functional, disability, and health data pertinent to the ICF. This encompasses a spectrum of data, including personal attributes, medical documentation, and functional evaluations. The aggregated data undergo rigorous cleansing, transformation, and standardization to be utilized effectively in the training and prediction phases of machine learning algorithms. In alignment with ICF directives, pertinent features are meticulously selected and engineered to enable algorithms to accurately discern and prognosticate functional, disability, and health statuses. This endeavor may encompass sophisticated techniques such as natural language processing and image recognition. Despite the challenges posed by the scarcity of comprehensive databases, it is incontrovertible that in the burgeoning era of big data and artificial intelligence, forensic science stands poised for unprecedented advancements across its various disciplines.

## 5. Conclusion

This study used XGBoost, LightGBM and Random Forest for modelling. In the real data set, XGBoost performed best, and the model accuracy rate was 66%. In the virtual data set, the performance of XGBoost and LightGBM was the same, and the model accuracy rate was 80%. The overall accuracy of external verification was 72.7%. The optimal model was expected to predict the degree of causality accurately. The model established this time can only be used to predict the size of the causal relationship of orthopaedic-related medical injuries. We have verified the feasibility of this method and can further establish prediction models for other departments in future studies.

## 6. Limitation

In this study, the real data set is imbalanced, especially for the label classification of "Whole", and the insufficient data volume reduces the model’s overall performance. The virtual data set formed by oversampling may have problems with overfitting and data leakage. The sample size can be increased, and the model can be optimised by undersampling. Only three decision tree model-based machine learning integration algorithms are used, and others can be tried. In addition, we can try to combine automatic text classification technology with machine learning to reduce the workload [53, 54]. This paper does not explain the specific algorithm of the model. How does the computer form the optimal model through the algorithm?

## Supporting information

S1 Table Modeling data set and feature set. (XLSX)

S1 File Machine learning code and best models. They are available at https://github.com/nerdyqx/ML.
(ZIP)

S2 File (DOCX)
